# Delayed interval delivery in a quadruplet pregnancy: a case report and literature review

**DOI:** 10.1186/s12884-023-05647-w

**Published:** 2023-05-15

**Authors:** Yanan Li, Ran Chu, Yarong Li, Meiling Zhang, Yuyan Ma

**Affiliations:** Department of Obstetrics and Gynecology, Qilu Hospital of Shandong University, 107 Wenhua Xi Road, Jinan, Shandong Province China

**Keywords:** Delayed interval delivery, Quadruplet pregnancy, Cervical cerclage, Case report

## Abstract

**Background:**

As the rate of multiple pregnancies increases, delayed interval delivery (DID) is increasingly being implemented to improve perinatal outcomes. But there are no international guidelines for DID in multiple pregnancies. We report a case of DID in a quadruplet pregnancy and review the relevant literature to summarize the management of DID in multiple pregnancies.

**Case presentation:**

A 22-year-old woman, 22 2/7 weeks' gestation, with quadruplets, was admitted to the hospital for a first cervical cerclage due to cervical dilation. Twenty-five days later, it was found that the cervix was dilated again, so after removing the cervical cerclage, the first quadruplet was delivered vaginally (25 6/7 weeks), and a second cervical cerclage was performed. Four days later, due to re-dilation of the cervix, after removal of the cervical cerclage, the second quadruplet was delivered vaginally (26 3/7 weeks), followed by a third cervical cerclage. Six days later, the pregnancy was terminated by cesarean section due to fetal distress, and the third and fourth quadruplets were delivered (27 2/7 weeks). The patient had no postoperative complications, and all four infants were treated in the neonatal intensive care unit and discharged successfully.

**Conclusion:**

This case emphasizes that comprehensive management of delayed interval delivery can improve perinatal outcomes in multiple pregnancies, including anti-infection, tocolytic therapy, practice to promote fetal lung, and cervical cerclage.

## Background

Delayed Interval Delivery (DID) refers to after the miscarriage or preterm delivery of one of the multiple gestations, the deliveries of other fetuses were delayed for at least 24 h to improve the perinatal outcome. In recent years, as assisted reproductive technologies have developed and become more widespread, the use of ovulation-inducing and super ovulatory drugs has increased, which has led to a significant increase in the multiple pregnancy rate [1]. The risk of fetal, neonatal and maternal complications is significantly higher in multiple pregnancies compared to singleton pregnancies. Preterm birth is the most common complication of multiple pregnancies [2]. DID in multiple pregnancies is increasingly being implemented in clinical practice to improve the perinatal outcomes of preterm neonates in multiple pregnancies [3].

Since international guidelines for DID in multiple pregnancies are still lacking, and the management of DID is based on expert experience and consensus, there is some controversy in the DID strategy, especially regarding whether to perform cervical cerclage. We herein report an uncommon case of successful delayed labor with good maternal and neonatal outcomes in a quadruplet pregnancy. We hope to provide valuable experience in the clinical management of quadruplet pregnancies with DID strategy.

## Case presentation

**Fig. 1:**
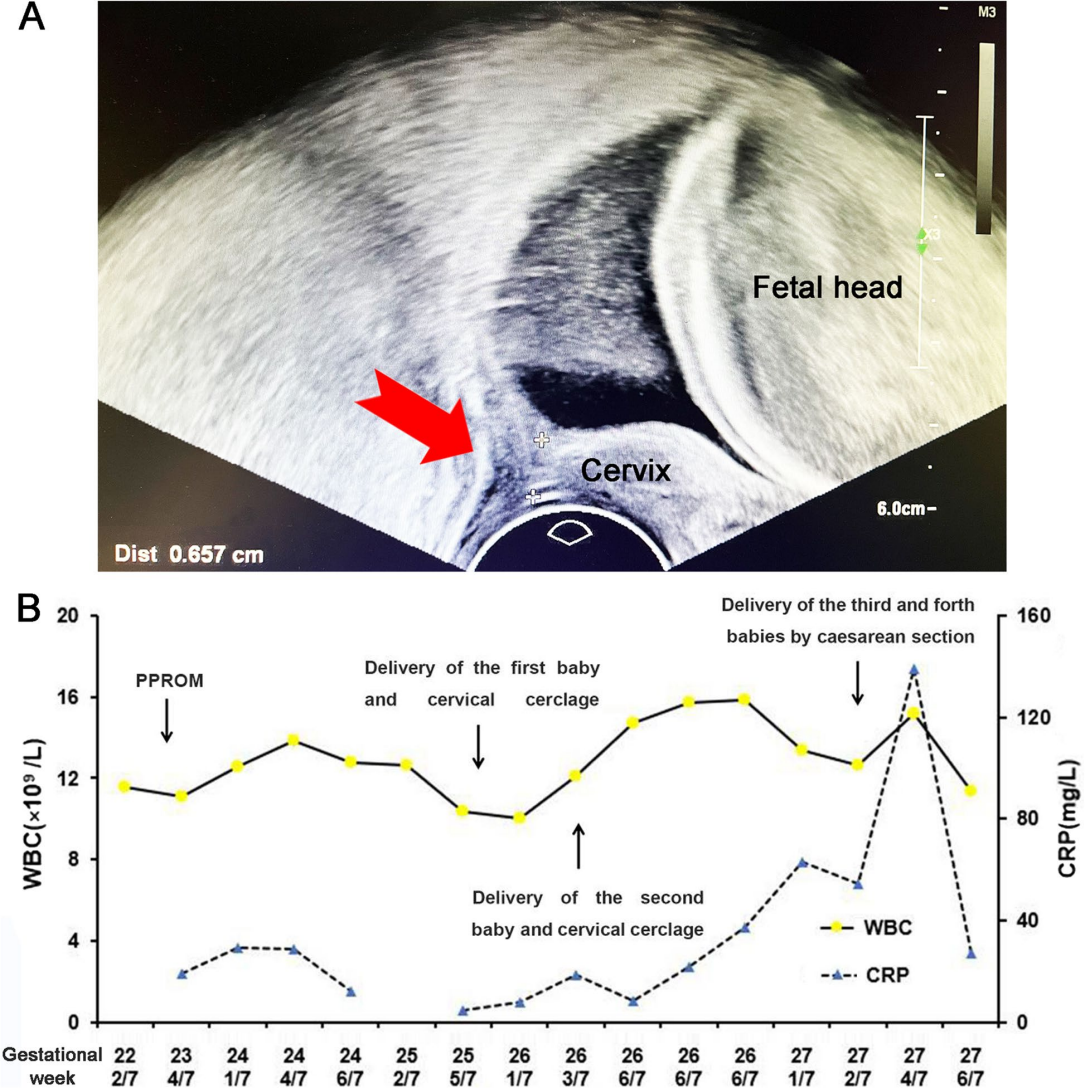
Ultrasound findings and time-trends in WBC and CRP values during hospitalization. **A** Ultrasound finds that the upper segment of the cervical canal was U-shaped, about 2 cm. The red arrow shows the closed cervical canal was only about 0.6 cm. **B** Time-trends in WBC (× 10^9^/L), solid line) and CRP (mg/L, dotted line) values during hospitalization. CRP: C-reactive protein; WBC: white blood cell; PPROM: preterm premature rupture of membranes

A 22-year-old woman, gravida 1, para 0, was admitted to the hospital at 22 2/7 weeks of quadruple pregnancy due to dilated cervix. The quadruple pregnancy was confirmed during the first trimester, following conception by ovulation promotion and she refused a reduction. She had three episodes of heavy vaginal bleeding during the first trimester and was treated at the local hospital. Ultrasound examination two days ago (22 weeks of pregnancy) found that the upper segment of the cervical canal was U-shaped, about 2 cm, the internal cervical was dilated, about 3.6 cm, and the closed cervical canal was only about 0.6 cm (Fig. 1A). The patient had no vaginal bleeding, liquor leaking, or tightness in the abdomen or uterus. Her vital signs were stable, and fetal monitoring was normal.

The patient was advised to stay strictly in bed and elevate the hips. Ritodrine hydrochloride was used to inhibit occasional uterine contraction and magnesium sulfate was used for neuroprotection. Ultrasonographic assessment again showed that the closed cervical canal was shorter than before and approximately 0.3 cm. The emergency transvaginal cervical cerclage was performed at 23 weeks of gestation under combined spinal-epidural anesthesia. The anterior amniotic sac protracted from the cervical orifice, with a small amount of amniotic fluid leakage. Postoperatively, we continued ritodrine hydrochloride, cefazoline and metronidazole were initiated because of elevated inflammatory markers to prolong the pregnancy. The surveillance protocol involved laboratory assessment (White Blood Cell [WBC] and C-Reactive Protein [CRP] levels three days a time), ultrasonographic evaluation twice a week, vital signs clinical assessment (arterial pressure, heart rate, temperature), and electronic fetal monitoring twice a day. The fluctuations of CRP and WBC values during the patient’s hospitalization are shown in the Fig. 1B.

At 23 3/7 weeks of pregnancy, a small amount of vaginal liquid was leaked, then it was confirmed via speculum examination, and suggesting amniotic fluid leakage. Laboratory examinations revealed the presence of mild elevated WBC (11.1*10^9/L) and CRP (18.94 mg/L with an upper normal laboratory limit of 10 mg/L). Chorioamnionitis was highly suspected, and amniocentesis was recommended to confirm the diagnosis. However, the patient declined the amniocentesis and requested to continue the pregnancy. Informed consent was obtained. At 25 4/7 weeks of pregnancy, the culture of vaginal discharge was suggestive of candida albicans infection, and clotrimazole vaginal tablets were given for intravaginal placement.

At 25 6/7 weeks of pregnancy, a large amount of streaky discharge was observed, and the cervical was dilated about 6 cm. Cervical cerclage was removed, and a male fetus was delivered, weighing 735 g, with Apgar scores of 6 at the first minute and 8 at 5 min, and transferred to the neonatal intensive care unit (NICU) for treatment. Then uterine contractions gradually decreased, and no signs of active labor were noticed. After informing the patient and family in detail of the possible benefits and complications, they chose to delay the delivery of other fetuses. Cervical cerclage was performed after high ligation of the umbilical cord. The vagina was rinsed with iodophor. We used ritodrine hydrochloride and indomethacin to prolong pregnancy and antibiotic therapy (cefazoline and metronidazole) was continued while continuously monitoring the patient and fetuses.

**Fig. 2:**
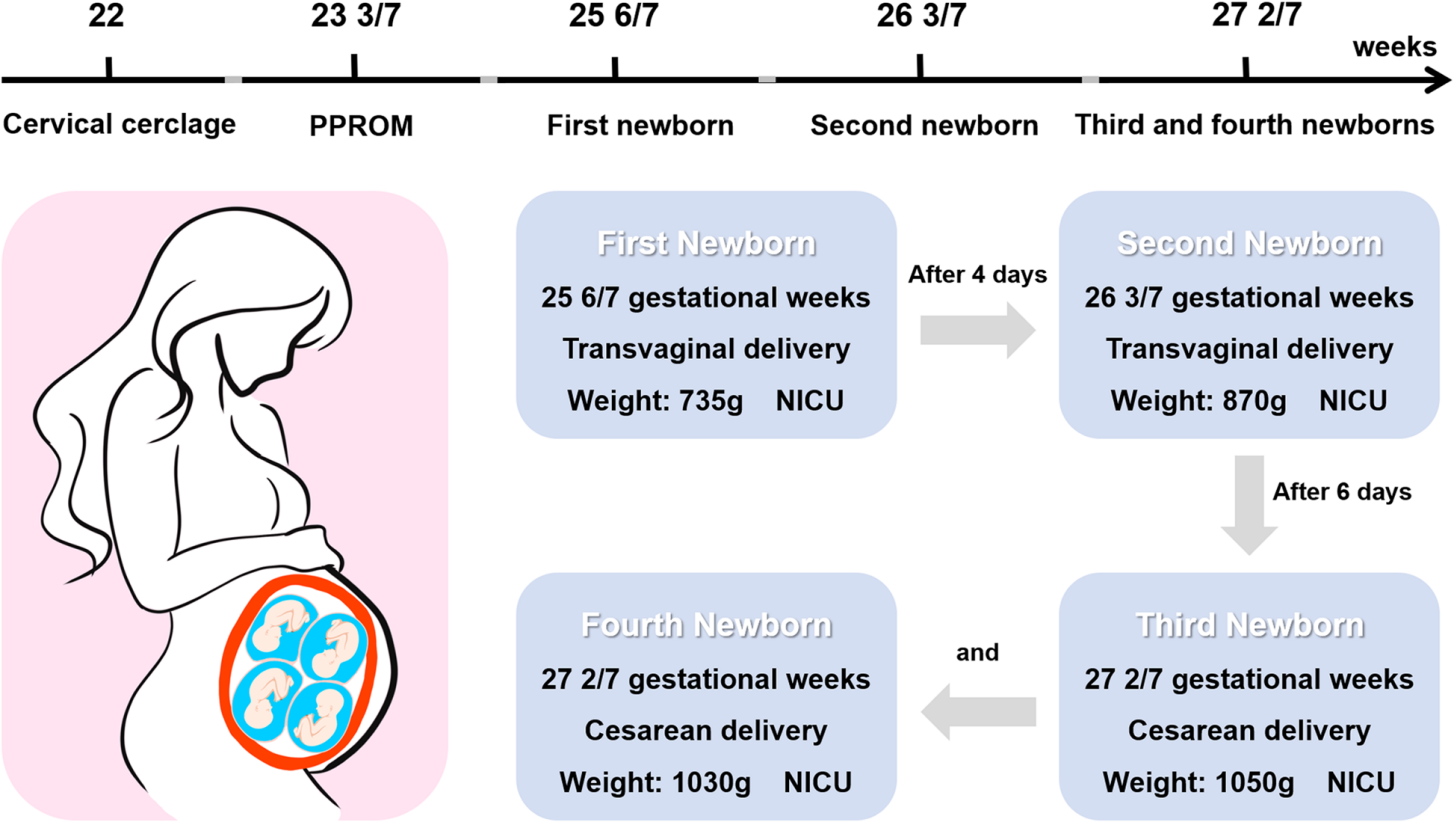
Flowchart of the case. PPROM: preterm premature rupture of membranes; NICU: neonatal intensive care unit. The image of Fig. 2 is drawn by ourselves

Four days later (26 3/7 weeks of pregnancy), there was a recurrence of vaginal discharge and irregular contractions. Ultrasound examination showed dilated cervical canal and part of the fetus protruding into the cervical canal. The fetal heart rate was normal. A second emergency cervical cerclage removal and transvaginal delivery of a fetus were performed. A second male infant was delivered, weighing 870 g, with Apgar scores of 7 at the first minute and 8 at 5 min, and was also transferred to the NICU. After careful consideration of the situation and wishes of the patient, DID has performed again. Given the progressively higher infection indicators and the more frequent uterine contractions, we started using meropenem to control the infection and atosiban to suppress contractions. Then, at 27 2/7 weeks of pregnancy (6 days after the last surgery), she had irregular uterine contractions with the outflow of amniotic fluid. Then, we observed a slowing of the fetal heart rate related to uterine contractions with gradual onset following the peak of the uterine contraction and delayed return of the deceleration to the baseline, usually following the contraction. The gradual decrease in fetal heart rate suggested fetal distress. After informed consent, the pregnancy was terminated by cesarean section. Two male infants were delivered, weighing 1050 g/1030 g, with Apgar scores of 7 at the first minute and 8 at 5 min both. The flowchart of the case is shown in Fig. 2.

**Fig. 3:**
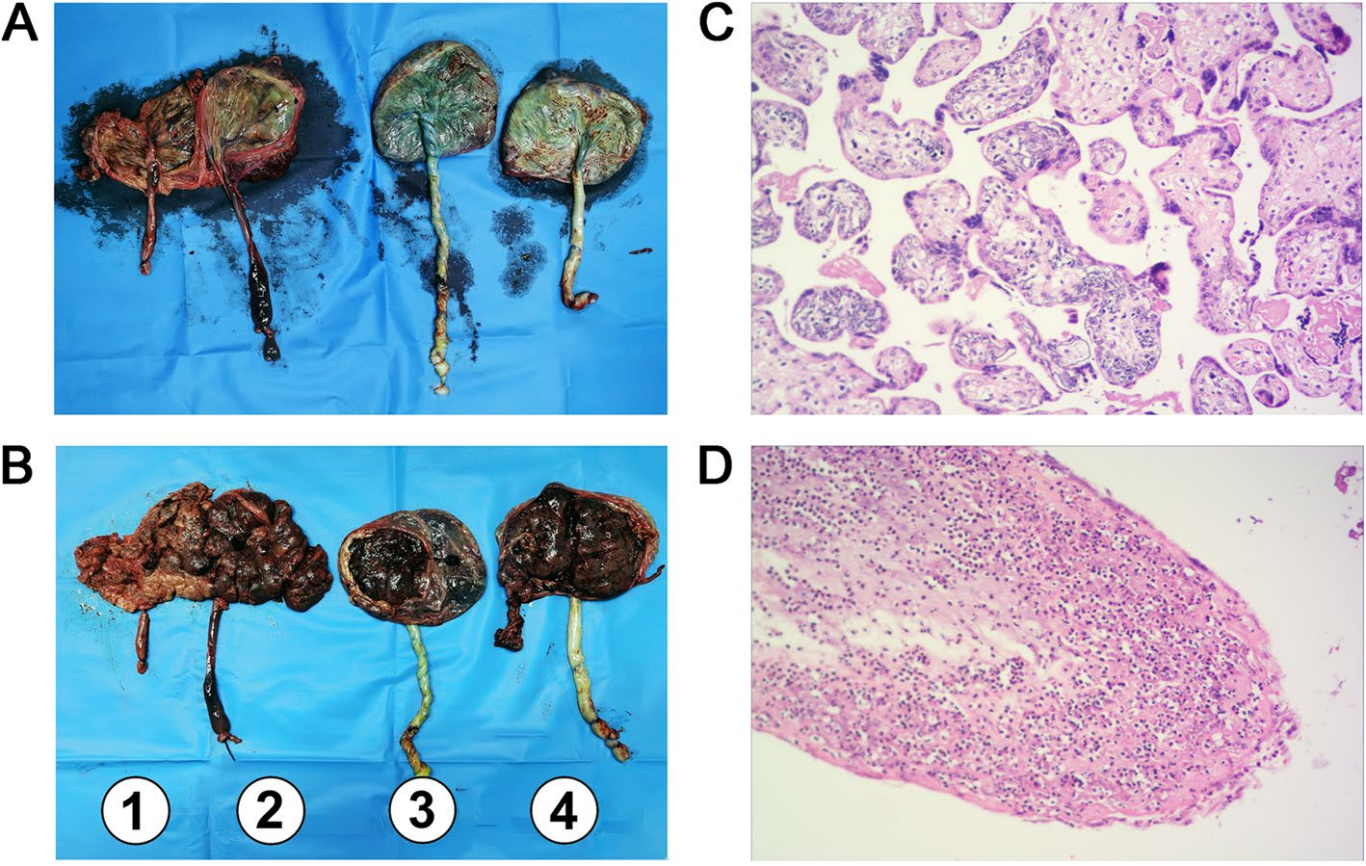
Fetal side (**A**) and maternal side (**B**) of four placentas; HE staining of placental tissue ([**C**] and [**D**]). (**A**) and (**B**): Placenta 1 and placenta 2 are fused, volume is about 25*8*2 cm. Placenta 3 is about 11*10.5*0.8 cm, and placenta 4 is about 11*9.5*3 cm. (**C**) and (**D**): HE × 100 and HE × 40. Amniotic epithelial cells are necrotic and detached. Continuously fused neutrophils are infiltrated, and some of these have fragmented nuclei

The patient continued on antibiotic (meropenem), anti-thrombotic (low molecular weight heparin) and uterotonic therapy (oxytocine) after the cesarean section. She remained afebrile although a pathological examination of the placenta found acute chorioamnionitis (Fig. 3). She was discharged four days after the operation without any complications.

Four preterm neonates were admitted to the NICU. They were all given meropenem to control infection, vitamin K1 to prevent bleeding, lung surface-active substances to promote fetal lung maturation and intravenous nutritional support, and antibiotics were adjusted according to infection and drug sensitivity. The first baby was also treated with ganciclovir for antiviral therapy and transfused with human immunoglobulin and platelets because of repeatedly low platelets. The second baby was also given creatine phosphate and levothyroxine sodium. They recovered well after neonatal treatment and were discharged successfully with hospital stays of 136, 92, 59, and 68 days respectively. All of them started to eat and parenteral feeding was stopped. All four babies had normal primitive reflexes and muscle tone. Serial electroencephalograms did not reveal any abnormalities in neurophysiological development.

## Discussion

As assisted reproductive technology continues to develop and become more widespread, the rate of multiple pregnancies has increased significantly, with 36% of twins and 77% of triplets and multiple births being the result of assisted conception [1]. Preterm birth is the most common complication of multiple pregnancies. The incidence of delivery before 32 weeks of gestation was 2%, 8%, 26%, and > 95% for singleton, twin, triplet, and quadruplet pregnancies, respectively [2]. In recent years, the gradual implementation of DID in multiple pregnancies has reduced the incidence of maternal and infant complications and effectively improved perinatal outcomes [4]. Only three cases of DID in quadruplet pregnancies have been reported worldwide [5–7]. Now we reported another similar case and we hope to be experienced in the management of DID in quadruplet pregnancies.

### Conditions for DID in multiple pregnancies

DID may be considered after delivery of the first child in multiple pregnancies if maternal uterine contractions are stopped, and the cervix reconstitutes. Contraindications for DID are fetal distress, congenital abnormalities, preterm rupture of membranes of the remaining fetus, chorioamnionitis, suspected placental abruption, and severe vaginal blood loss. Gestational age and chorionicity suitable for DID are controversial.

The minimum gestational age reported for the first delivery of DID is 15 3/7 weeks, and the second fetus was delivered at 31 weeks of gestation [8]. Benden et al. [9] suggest that the lower limit of gestational weeks for the delivery of the first fetus for DID is 20–22 weeks, and the upper limit can be 28 weeks of gestation. Based on the high neonatal mortality and morbidity rates associated with deliveries before 32 weeks of pregnancy, the maximum gestational age for delayed labor is considered to be 31 weeks according to Rosbergen [10]. Porreco and Farkouh [11] suggested that DID may be considered if the first fetus is delivered before 24 weeks gestation, because preterm and aborted fetuses delivered at ≤ 24 weeks of gestational age have little chance of survival, but the fetus is more likely to survive if DID is used to preserve the fetus beyond 24 weeks of gestation. But for fetuses born at 28 weeks of gestational, DID is not recommended because they usually survive and have a good outcome depending on the treatment of the NICU. Otherwise, DID may lead to maternal and fetal complications, such as placental abruption, severe preeclampsia and chorioamnionitis, which can lead to poor outcomes. A meta-analysis assessing mortality in the second twin when the first was delivered at ≥ 24 weeks found a significant reduction in the second twin’s mortality [12]. To sum up, although there are currently no standard guidelines, we can find that almost all studies support that if the first fetus is delivered at less than 28 weeks gestational age and there are no contraindications, DID can be considered. The maternal and intrauterine fetal conditions and the level of NICU treatment should be comprehensively assessed to achieve better perinatal outcomes before the implementation of DID in multiple pregnancy.

Most cases reported in the available literature are DID in dichorionic-diamniotic twin pregnancies. It is currently considered that DID is considered contraindicated in monochorionic twins because of the increased risk of chorioamnionitis and placental vascular anastomosis between the two fetuses, which may lead to severe complications in the undelivered fetus. However, there have been a few case reports of successful DID in monochorionic diamniotic twin pregnancy [13]. Therefore, whether monochorionic is a contraindication to DID in multiple pregnancies needs further exploration and confirmation.

In our case, the quadruplet was four chorions and amniotic sacs, and the first fetus was delivered at 25 6/7 weeks of gestation. So, we performed DID on this quadruple pregnancy after the exclusion of contraindications. The second fetus was born four days later, and the third and fourth fetuses were delivered ten days later. All four infants were generally stable after neonatal treatment and the length of hospitalization was significantly shorter for the third and fourth infants, demonstrating that implementation of DID after the exclusion of contraindications effectively improves neonates' prognosis in multiple gestations.

### Management of DID in multiple pregnancies

#### Prevention of infection

One of the prerequisites for successful DID in multiple pregnancies is the exclusion of maternal chorioamnionitis. DID can lead to poor maternal and neonatal outcomes once infection occurs. Most scholars now suggest that after delivery of the first fetus in multiple pregnancies, the vagina should be strictly disinfected and the umbilical cord should be ligated in a high position with absorbable sutures [3, 14]. The patient should be treated with broad-spectrum antibiotics for 72 h. After that, oral antibacterial drugs should be continued for seven days according to the results of the cervical and vaginal secretion culture [15–17]. Graham et al. [18] recommend the prophylactic use of broad-spectrum antibiotics such as ampicillin, sulbactam and metronidazole to cover a wide range of bacteria isolated from the cervix, placenta and amniotic fluid in the setting of DID, with cervical and vaginal secretion culture repeated once a week and antibiotic use adjusted according to culture results.

In our case, the patient had a preterm premature rupture of membranes, maternal laboratory examinations were suggestive of the presence of infection, but there were no clinical signs of uterine tenderness or fever. The patient was informed of the possible presence of chorioamnionitis and amniocentesis was advised to rule out infection. However, she declined the operation and wished to continue her pregnancy with conservative treatment. WBCs and CRP were routinely assessed every three days. Broad-spectrum antibiotics were used to treat infection until the delivery of the fourth child. A culture of vaginal secretions suggested Candida albicans infection and intravaginal clotrimazole was placed to prevent the further development of the infection and prolong the DID interval. Therefore, the prophylactic use of broad-spectrum antibiotics can effectively prevent and treat infection, prolong the birth interval and improve perinatal outcomes for both mother and baby.

#### Tocolytic therapy

The use of tocolytic in DID is controversial, and practice patterns among specialists vary widely. There are insufficient data to support or refute the use of tocolytic therapy.

The tocolytics such as β2 agonists, calcium channel blockers, magnesium sulfate, prostaglandin synthase inhibitors and atosiban can be used prophylactically or therapeutically after delivery of the first fetus. However, there are no uniform standards for the type or dosage of tocolytics for DID. For patients with signs of preterm labor, prostaglandin synthase inhibitors and calcium channel blockers are preferred after a comprehensive assessment of four dimensions: gestational age, neonatal mortality, neonatal respiratory distress syndrome, and maternal side effects [19]. Magnesium sulfate or indomethacin have also been proposed to use in DID [14], but anti-inflammatory drugs may mask the symptoms of chorioamnionitis [18]. Few cases reported the combination of tocolytics, but it may further increase maternal side effects, and the combination's safety needs to be supported by further clinical data.

#### Cervical cerclage

The most controversial issue in DID is cervical cerclage. It has been argued that cervical cerclage closes the cervical canal, reduces the risk of upstream infection, and increases cervical stability and support for the retained fetus in utero. Conversely, it has also been suggested that the invasive technique may increase the risk of chorioamnionitis. Zhang et al. [20] proposed that cervical cerclage immediately after the first delivery can prolong the interval between deliveries by reducing the exposure of the membranes to the bacterial and acidic environment of the vagina, without increasing the risk of intrauterine infection. Fayad et al. [21] observed that the mean delayed interval between births was longer after cervical cerclage, but the difference was not statistically significant, suggesting that cervical cerclage did not significantly affect the delayed duration of labor. Farkouh et al. [15] reported that the latency period was significantly shorter in patients with a history of cervical cerclage than in those without. Berghella et al. [22] found a higher incidence of preterm labor with cervical cerclage in twin pregnancies. The application of cervical cerclage in DID remains a highly controversial issue, and the balance between upstream infection and support stability is difficult to manage. In clinical practice, the choice should be made based on the patient’s situation and the personal experience of the obstetrician.

In our case of a quadruplet pregnancy, the cervical canal was already shortened at 23 2/7 weeks of gestation due to high intrauterine pressure. To avoid the abortion, cervical cerclage was performed before delivery of the first fetus, significantly prolonging the interval and improving the survival rate. After delivery of the first and the second fetus, cervical cerclage was performed respectively, and postoperative antibiotics were used to prevent infection. Vaginal discharge was regularly tested, and antibiotics were targeted. Tocolytics were used in combination. In the presence of high intrauterine pressure, cervical cerclage effectively increased the stability of the cervix and improved support for the retained fetus, delaying delivery by 4 and 6 days, respectively, with significant improvement in neonatal outcome and a good maternal recovery with no significant complications. Because cervical insufficiency cannot usually be ruled out as a contributing factor to the preterm labor of the first fetus, we generally perform cervical cerclage in patients accepting DID, which can temporarily prevent delivery of the retained intrauterine fetus. Even if cervical insufficiency was not the initial contributing factor, cervical cerclage after delivery of the first fetus can prevent detachment of the fetal membranes and reduce the risk of amniotic cavity infection.

#### Practice to promote fetal lung

Some scholars advocate that glucocorticoids should be used to promote fetal lung when the gestational age of the delayed delivery fetus is extended to 24 weeks, [23] but some scholars recommend that glucocorticoids should be used after 26 weeks of gestation [21, 24]. Porreco and Farkouh [11] differ slightly in their recommendations regarding the timing of administration, with a course of glucocorticoids being given for fetuses over 23 weeks of gestational age, but not for fetuses younger. Because fetuses younger than 23 weeks of gestational age have only a minimal number of primitive alveoli, glucocorticoids do not promote fetal lung maturation and increase the risk of adverse effects. After two weeks of the first course of glucocorticoids for fetal lung maturation, a second course of treatment may be given if the remaining fetus shows signs of preterm labor.

### Maternal and neonatal outcomes

#### Maternal outcomes

Adverse maternal outcomes are mainly associated with the development of infectious diseases [14, 18], with the literature reporting intrauterine infections in 36% of delayed deliveries and sepsis in 4. 9% [14]. Arabin et al. [25] found that common maternal complications were chorioamnionitis (22%), postpartum hemorrhage (10%), retained placenta (10%), and abruption placenta (6%).

In this case, the patient's laboratory examinations were initially suggestive of the presence of infection, but no symptoms occurred. The postoperative placental pathology showed chorioamnionitis, but the patient recovered well after delivery without complications. It may be closely related to our anti-infective treatment during pregnancy.

#### Neonatal outcomes

The perinatal morbidity of DID fetuses and the incidence of complications depend largely on the gestational age. The literature reports that the interval between deliveries of fetuses in multiple pregnancies varies from 1 to 152 days, with a 3% increase in neonatal survival for each day of delay between 23 and 26 weeks of gestation [23]. The survival rate of the second fetus is reported to be around 78% in some cases [26]. Perinatal complications are predominantly respiratory diseases and infections. In a study by Roman of a total of 16 live births, 12 (75%) had respiratory distress syndrome, 2 (12.5%) had pulmonary bronchiectasis and 6 (37.5%) had severe infections [14]. The survival rate of delayed fetal is higher, and adverse outcomes and morbidity are reduced. Infants with delayed delivery have good short-term outcomes and long-term outcomes compared to those of the same gestational age [26].

In recent years, the prognosis for preterm neonates has significantly improved thanks to NICU treatment improvements. The length of hospitalization was 136, 92, 59, and 68 days, respectively, and all were discharged successfully after their general condition was stable, with further follow-up for long-term complications.

## Conclusion

Given the complications of multiple pregnancies and the burden of it on families and the country, the management of assisted reproductive technology to reduce the incidence of multiple pregnancies should be strengthened. Higher-order pregnancies should be prevented by reducing the number of embryos during transfer and better monitoring during ovulation induction in fertility treatment. A quadruplet pregnancy is high-risk, and the pregnant woman should be advised to undergo a reduction in the first trimester. In case of a miscarriage or preterm labor in continuing pregnancy, DID may be an option after excluding relevant contraindications. After delivery of the first fetus, the umbilical cord should be ligated in a high position, and given anti-infection, tocolytics and dexamethasone. Cervical cerclage can be performed to prolong the delayed interval. There are no consensus guidelines for managing DID in multiple pregnancies, and it is important to consider the maternal condition and the level of obstetric and neonatal care to choose the best management and improve perinatal outcomes.

## Data Availability

Data are available at Qilu Hospital of Shandong University archives and can be sent by the corresponding author on request.

## Abbreviations

CRP: C-reactive protein
DID: Delayed interval delivery
NICU: Neonatal intensive care unit
WBC: White Blood Cell
